# PseU-ST: A new stacked ensemble-learning method for identifying RNA pseudouridine sites

**DOI:** 10.3389/fgene.2023.1121694

**Published:** 2023-01-19

**Authors:** Xinru Zhang, Shutao Wang, Lina Xie, Yuhui Zhu

**Affiliations:** Department of Pharmacy, The Second Hospital of Jilin University, Changchun, China

**Keywords:** RNA pseudouridine site identification, sequence analysis, computational methods, machine learning, stacked ensemble-learning

## Abstract

**Background:** Pseudouridine (Ψ) is one of the most abundant RNA modifications found in a variety of RNA types, and it plays a significant role in many biological processes. The key to studying the various biochemical functions and mechanisms of Ψ is to identify the Ψ sites. However, identifying Ψ sites using experimental methods is time-consuming and expensive. Therefore, it is necessary to develop computational methods that can accurately predict Ψ sites based on RNA sequence information.

**Methods:** In this study, we proposed a new model called PseU-ST to identify Ψ sites in *Homo sapiens (H. sapiens)*, *Saccharomyces cerevisiae (S. cerevisiae)*, and *Mus musculus (M. musculus)*. We selected the best six encoding schemes and four machine learning algorithms based on a comprehensive test of almost all of the RNA sequence encoding schemes available in the iLearnPlus software package, and selected the optimal features for each encoding scheme using chi-square and incremental feature selection algorithms. Then, we selected the optimal feature combination and the best base-classifier combination for each species through an extensive performance comparison and employed a stacking strategy to build the predictive model.

**Results:** The results demonstrated that PseU-ST achieved better prediction performance compared with other existing models. The PseU-ST accuracy scores were 93.64%, 87.74%, and 89.64% on H_990, S_628, and M_944, respectively, representing increments of 13.94%, 6.05%, and 0.26%, respectively, higher than the best existing methods on the same benchmark training datasets.

**Conclusion:** The data indicate that PseU-ST is a very competitive prediction model for identifying RNA Ψ sites in *H. sapiens*, *M. musculus*, and *S. cerevisiae*. In addition, we found that the Position-specific trinucleotide propensity based on single strand (PSTNPss) and Position-specific of three nucleotides (PS3) features play an important role in Ψ site identification. The source code for PseU-ST and the data are obtainable in our GitHub repository (https://github.com/jluzhangxinrubio/PseU-ST).

## 1 Introduction

Pseudouridine (Ψ) is one of the most abundant RNA modifications found in many RNAs, such as rRNA, mRNA, tRNA, and snRNA et al. (Charette and Gray, 2000). Research on Ψ has been developing since its discovery in 1957. Many studies have shown that Ψ plays a key role in several bioprocesses, including the maintenance of RNA construction stability (Boo and Kim, 2020), the metabolism of RNA (Carlile et al., 2014; Schwartz et al., 2014), and the RNA-protein or RNA-RNA interactions (Basak and Query, 2014). Previous studies also found that Ψ mutations are related to many cancers, such us lung and stomach cancer (Itoh et al., 1989; Penzo et al., 2017; Cao et al., 2021). The key to studying the various biochemical functions and mechanisms of Ψ is to identify the Ψ sites. However, identifying Ψ sites using experimental methods is time-consuming and expensive (Adachi et al., 2019). Therefore, it is necessary to develop computational methods which can accurately predict Ψ sites based on the RNA sequence information.

In recent years, many computational predictors of Ψ sites have been developed to complement experimental studies. Li et al. (2015) established the first computational model to predict Ψ sites in *S. cerevisiae* and *H. sapiens,* named PPUS, using support vector machine (SVM) algorithms. Similarly, Chen et al. (2016) established a SVM model called iRNA-PseU by combining the encoding schemes of pseudo-nucleotide composition and nucleotide chemical property (NCP) to predict Ψ sites in 2016. Subsequently, He et al. (2018) developed another SVM classifier called PseUI, which extracts RNA sequence features using five different encoding schemes. Tahir et al. (2019) established a convolutional neural network (CNN) model, named iPseU-CNN, which employs the binary encoding scheme. In 2020, Liu et al. (2020) proposed XG-PseU using eXtreme Gradient Boosting (XGBoost) algorithms to predict Ψ sites. In the same year, Bi et al. (2020) created an ensemble model called EnsemPseU, which integrates random forest (RF),SVM, Naïve Bayes (NB), XGBoost, and k-nearest neighbours (KNN). Lv et al. (2020) developed an RF-based method called RF-PseU, which applies a light gradient boosting machine (lightGBM) algorithms to identify Ψ sites. Mu et al. (2020) presented a layered ensemble model designated as iPseU-Layer, which applies classic RF to predict Ψ sites. Then, Li et al. (2021b) proposed a computational model called Porpoise, which selects four optimal types of features and fed them into a stacked model to predict Ψ sites. Zhuang et al. (2021) proposed PseUdeep, a deep learning framework, and Wang et al. (2021) proposed a feature fusion predictor named PsoEL-PseU in the same year; however, their performance are unsatisfactory. The accuracy scores of the best existing methods mentioned above are 79.70%, 81.69%, and 89.34% in *H. sapiens*, *S. cerevisiae*, and *M. musculus*, respectively, so there is still much opportunity for improvement.

In this study, we proposed a new model called PseU-ST to identify Ψ sites in *H. sapiens, S. cerevisiae*, and *M. musculus*. First, we thoroughly tested almost all of the available RNA sequence encoding schemes in the iLearnPlus software package with seven most popular machine learning algorithms and selected the best six types of encoding schemes and four machine learning algorithms (Cui et al., 2022). We then sorted the feature importance of the six encoding schemes separately using chi-square and selected the optimal features for each encoding scheme using incremental feature selection (IFS) algorithms. We used the cross-validation tests to evaluate and select the optimal feature and base-classifier combinations for each species. Next, we employed a stacking strategy to establish a predictive model. The results demonstrated that PseU-ST achieved better prediction performance compared with other existing models. Therefore, PseU-ST is a highly competitive prediction model for identifying RNA Ψ sites in *H. sapiens, S. cerevisiae*, and *M. musculus*.

## 2 Materials and methods

### 2.1 The framework of PseU-ST

**FIGURE 1:**
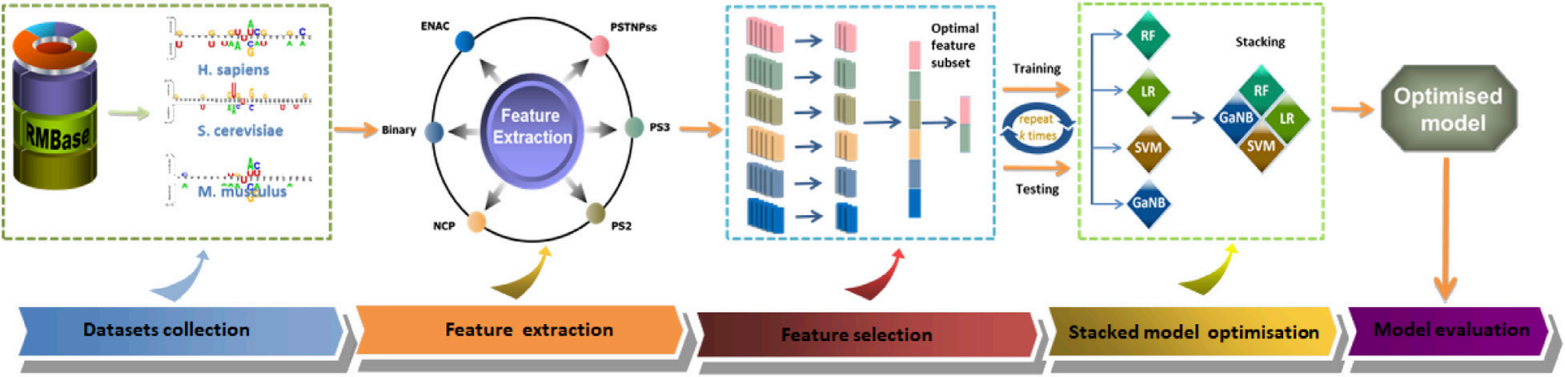
The overall framework of PseU-ST. There are five major steps, including dataset collection, feature extraction, feature selection, stacked model optimisation, and model evaluation.

The general framework design of PseU-ST is shown in Figure 1. The framework of PseU-ST had five major steps. Step 1, we saved the training datasets and the independent test datasets from online databases (Chen et al., 2016). Step 2, we thoroughly tested almost all of the available RNA sequence encoding schemes in the iLearnPlus software package with seven most popular machine learning algorithms and selected the best six encoding schemes and four algorithms. Step 3, we sorted the feature importance of the six encoding schemes separately using chi-square and selected the optimal features for each encoding scheme using IFS algorithms. We then built models using different combinations of optimal features and selected the optimal feature combinations for each species. Step 4, we built RF, SVM, Gaussian Naive Bayes (GaNB), and logistic regression (LR) models separately using the optimal feature combination selected in the forward step as the preliminary base-classifier; LR was used as the meta-classifier, and we built a series of stacked models by using different base-classifier combinations and selected the best base-classifier combination for each species. Step 5, we compared the predictive performance of the optimised stacked model in 5-fold cross-validation and independent tests with those of other existing models.

### 2.2 Dataset collection

Chen et al. (Chen et al., 2016) collected datasets from RMBase (Sun et al., 2016) to identify Ψ sites by machine learning methods. First, RNA fragments with uridine (U) in the center were collected by sliding the (2ξ + 1)-tuple nucleotide window along the RNA sequences; when the center of RNA sample is confirmed as Ψ site by experiment, it is considered positive, otherwise it is negative. Then, the samples with ≥60% paired sequence identity were screened out with any other samples in the same class using CD-HIT software, and the negative and positive subsets were made to have the same size using a random-picking procedure. The training datasets contained three datasets, they were H_990 (*H. sapiens*), M_944 (*M. musculus*), and S_628 (*S. cerevisiae*), while there were only two species, namely H_200 (*H. sapiens*) and S_200 (*S. cerevisiae*) in the independent testing datasets. Both the training and independent testing datasets had half-positive and half-negative samples. In addition, Chen et al. evaluated the performance of the predictor in identifying Ψ sites with different ξ values and found that when ξ = 10, the accuracy of *H. sapiens* or *M. musculus* reached a peak value, whereas that of *S. cerevisiae* reached a peak value when ξ = 15. Thus, the RNA sequence lengths in H_990 and M_944 were both 21 nt, and that in S_628 was 31 nt. The RNA sequence lengths in H_200 and S_200 were 21 and 31 nt, respectively. In recent years, the models mentioned in the introduction have all used the same datasets. In our study, we built the PseU-ST models using the same datasets. Detailed information on these datasets is presented in Table 1. Benchmark datasets were downloaded from http://lin-group.cn/server/iRNAPseu/data *.*

**TABLE 1:** Training and independent dataset information.

Species	Datasets	Length (bp)	Positive samples	Negative samples
*H. sapiens*	H_990 (training)	21	495	495
	H_200 (testing)	21	100	100
*S. cerevisiae*	S_628 (training)	31	314	314
	S_200 (testing)	31	100	100
*M. musculus*	M_44 (training)	21	472	472
	—	—	—	—

### 2.3 Feature extraction

In the computational model construction, feature extraction is a critical step. In our study, we thoroughly tested almost all of the available RNA sequence encoding schemes in the iLearnPlus software package (Chen et al., 2021). Then, according to their predictive performance, the best six encoding schemes were selected to determine the optimal feature combinations, including enhanced nucleic acid composition (ENAC), binary features, NCP, position-specific trinucleotide propensity based on single-strand (PSTNPss), position-specific of two nucleotides (PS2), and position-specific of three nucleotides (PS3) (Chen et al., 2017).

#### 2.3.1 Enhanced nucleic acid composition

ENAC calculates the nucleic acid composition based on fixed length window (the default value is 5) of the sequence, the window slides from the 5′end of the RNA sequence to the 3′ end continuously, and encodes the RNA sequence into equal length feature vectors.

#### 2.3.2 Binary feature (also called one-hot)

In binary encoding, four-dimensional binary vectors are used to represent nucleotides, for example, the A, C, G, and U in RNA are encoded to (1 0 0 0), (0 1 0 0), (0 0 1 0), and (0 0 0 1), respectively.

#### 2.3.3 Nucleotide chemical property

According to the differences of chemical bonds and chemical structures, the four nucleotides of RNA sequences (ACGU) are classified into three different classes, as shown in Table 2.

**TABLE 2:** Chemical structure of each nucleotide (Chen et al., 2015).

Chemical property	Class	Nucleotides
Ring Structure	Purine	A, G
	Pyrimidine	C, U
Functional Group	Amino	A, C
	Keto	G, U
Hydrogen Bond	Strong	C, G
	Weak	A, U

Based on their different chemical properties, we can use three-dimensional coordinates to encode A, C, G, and U, they are encoded as (1,1,1), (0,0,1), (0,1,0), and (1,0,0), respectively.

#### 2.3.4 Position-specific trinucleotide propensity based on single strand

The PSTNPss encodes DNA or RNA sequences using statistical rule. Generally, there were 4^3^ (i.e. 64) trinucleotides, for example, AAA, AAC, AAG, UUU (TTT). Thus, for a given RNA sequence of L-bp length, the position specificity of trinucleotide is defined as a 64 × (*L*-2) Matrix:

$$Z=\left[\begin{array}{ccc} Z_{1,1}\;Z_{1,2} & \cdots & Z_{1,L-2}\\ Z_{2,1}\;Z_{2,2} & \cdots & Z_{2,L-2}\\ \vdots \vdots & \ddots & \vdots \\ Z_{64,1}\;Z_{64,2} & \cdots & Z_{64,L-2} \end{array}\right]$$ (1)

where

$$Z_{i,j}=F^{+}\left({3mer}_{i}|\;j\right)-F^{-}\left({3mer}_{i}|\;j\right),\;i=1,2,\ldots ,64;j=1,2,\ldots ,L-2$$ (2)

F^+^(3mer _*i*_ *|j*) and F^−^ (3mer _*i*_ *|j*) respectively indicate the occurrence frequency of the *i*th trinucleotide (3mer_*i*_) at the *j*th position in the positive (S^+^) and negative (S^−^) datasets, and where 3mer_*1*_ = AAA, 3mer_*2*_ = AAC, and 3mer_*64*_ = UUU. Thus, an *L*-bp-long RNA sequence is denoted as:

$${S=\left[\emptyset_{1},\emptyset_{2},\ldots ,\emptyset_{L-2}\right]}^{T}$$ (3)

where T is the transpose operator and ∅_*u*_ is expressed as:

$$\emptyset_{u}=\left\{\begin{array}{c} Z_{1,u\;},\;when\;N_{u}N_{u+1}N_{u+2}=AAA\\ Z_{2,u}\;,\;when\;N_{u}N_{u+1}N_{u+2}=AAG\;\\ \vdots \\ Z_{64,u\;},\;when\;N_{u}N_{u+1}N_{u+2}=UUU \end{array}\right.$$ (4)

Thus, in our study, the samples are denoted by 21–2 = 19 PSTNPss features in H_990 and M_944, and the samples are coded by 31–2 = 29 PSTNPss features in S_628.

#### 2.3.5 Position-specific of two nucleotides (PS2) and position-specific of three nucleotides (PS3)

There are 16 (i.e. 4 × 4) pairs of adjacent paired nucleotides, e.g. AA/AT/AG ...; therefore, a single variable representing such a paired nucleotide can be encoded as 16 binary variables and becomes binary. For example, AA is expressed as (1000000000000000), AC is (0100000000000000) …, and AAC is (10000000000000000100000000000000). PS3 is encoded by three adjacent nucleotides (4 × 4 × 4 = 64) in a similar manner.

### 2.4 Feature selection

A helpful method to remove redundancy and avoid over-fitting in computational modelling is feature selection as it plays a crucial role in improving the model performance (Jones et al., 2021; Suresh et al., 2022). To effectively represent sequences, in this study, we first sorted the feature importance of the six encoding schemes separately using a chi-square test and selected the optimal feature set for each of them using IFS algorithms (Lv et al., 2020; Zhang et al., 2021). Subsequently, we determined the optimal feature combinations. We trained the optimal features of the six encoding schemes using the best four algorithms selected in the stacking ensemble learning model section and ranked them according to accuracy (ACC). Then, we used the first-ranked feature to build the PseU-ST model, added the second feature to build a new model, and then added the third feature until all obtained features were added. Finally, we selected the optimal feature combinations for each species.

### 2.5 Stacking ensemble learning models

The stacking strategy can combine information from multiple classifiers to generate a more stable stacking model. It is a very useful integrated learning method that has been successfully applied to bioinformatics (Mishra et al., 2019; Li et al., 2021a). The “*mlxtend*” package in python (Raschka, 2018) provides a stacking cross-validation algorithm, which prepares input data for meta-level classifier by extending the standard stacking cross-validation algorithm. Moreover. The stacking strategy can be implemented using this algorithm. The stacking strategy can minimise the generalisation error rate of several predictive models (Su et al., 2020) and effectively avoids over-fitting (Sherwani et al., 2021). In this study, we employed a stacking strategy to establish a predictive model for RNAΨ sites. The stacking learning strategy has two major steps. Step 1, we built a series of classifiers, called base-classifiers. Step 2, we used the outputs obtained in the previous step of the base-classifiers as the input to train another classifier, called meta-classifiers.

In our study, we assessed the seven most popular algorithms: RF, LR, SVM, GaNB, Adaptive Boosting (AdaBoost), XGBoost, and Gradient Boosting Decision Tree (GBDT). RF is an integrated learning algorithm based on a decision tree. It can obtain accurate and stable predictions by building multiple decision trees and merging them. RF is one of the commonly used algorithms in bioinformatics (Lv et al., 2020; El Allali et al., 2021; Yin et al., 2021). LR is a generalised linear classification algorithm, it uses the *sigmod* function for non-linear mapping of all data to limit the prediction value to [0,1] and reduces the prediction range to classify samples. LR is a common machine learning method (Wei et al., 2020; Li and Wang, 2021; Zhu et al., 2021). SVM is another linear classification algorithm that is one of the most popular algorithms in computational biology (Chen et al., 2016; He et al., 2018). The decision boundary of SVM is to find an optimal separating hyperplane to segment samples. GaNB classifies sample data using probability and statistical methods based on the Bayesian theorem, assuming that the feature conditions are independent of each other. GaNB is also a commonly used algorithm (Yan et al., 2020; Shah et al., 2022). AdaBoost, XGBoost, and GBDT are all boosting models. They learn using different methods and form a strong classifier. They are widely used in bioinformatics (Liu et al., 2020; El Allali et al., 2021; Jayashree et al., 2022; Niu et al., 2022).

For each algorithm, we selected default parameters for training. For example, we set the tree numbers as 100 and the tree range as 100:1000:100 for RF. For SVM, the kernel function selected rbf, the penalty parameter selected 1.0, and the penalty range and gamma range was 1.0:15.0:1.0 and −10.0:5.0:1.0, respectively. For XGBoost, the booster parameter selected gbtree, the max depth was set as 3, and the penalty range was 3:10:1. Based on these parameters, we selected the best four algorithms for training the stacked models through an extensive performance comparison. Subsequently, we trained the optimal feature combinations of the three species that were previously determined using the best four algorithms as the candidate base classifier. We trained the stacked models using LR as the meta-classifier, and we evaluated the different combinations of base classifiers to select the best base-classifier combination as the final model.

### 2.6 Evaluation metrics

We used several widely used performance metrics to evaluate and compare the function of PseU-ST and other existing methods. The metrics are sensitivity (Sn), specificity (Sp), accuracy (ACC), Matthew’s Correlation Coefficient (MCC), and area under the receiver operating curve (AUC) (Mu et al., 2020; Li et al., 2021a; Zhuang et al., 2021). Sn, Sp, ACC, and MCC are defined as follows:

$$Sn=\frac{TP}{TP+FN}$$ (5)

$$Sp=\frac{TN}{FP+TN}$$ (6)

$$ACC=\frac{TP+TN}{TP+TN+FP+FN}$$ (7)

$$MCC=\frac{TP\times TN-FP\times FN}{\sqrt{\left(TP+FP\right)\times \left(TP+FN\right)\times \left(TN+FP\right)\times \left(TN+FN\right)}}$$ (8)

where TP, TN, FP, and FN represent the true positive, true negative, false positive, and false negative, respectively. We drew receiver operating characteristic (ROC) curves with 1-Sp as abscissa and Sn as ordinate and calculated AUC values.

## 3 Results and discussion

### 3.1 Determine the optimal feature combinations

**FIGURE 2:**
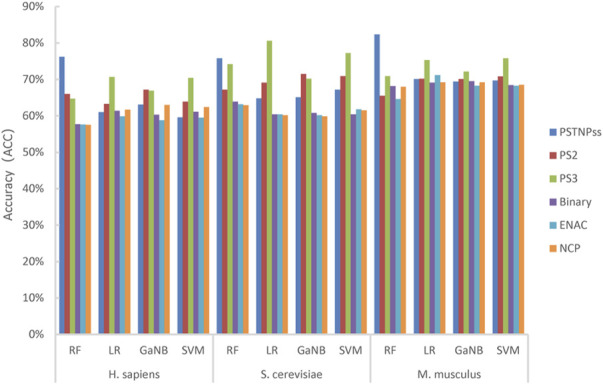
The accuracy of the four models trained using the best six encoding schemes for *H. sapiens*, *S. cerevisiae*, and *M. musculus*.

First, we thoroughly tested almost all of the RNA sequence encoding schemes available in the iLearnPlus software package with seven widely used machine learning algorithms, and built models for each algorithm with default parameters. Then, the best six encoding schemes and four machine learning algorithms were selected to build the stacked models. The best six encoding schemes were ENAC, binary feature, NCP, PSTNPss, PS2, and PS3, and the best four algorithms were LR, RF, SVM, and GaNB. For each algorithm, we trained six separate classifier features and ranked them according to the ACC. The ACC of each model is shown in Figure 2.

As shown in Figure 2, RF achieved the highest ACC for H_990 and M_944, whereas LR reached the highest ACC for S_628. The PSTNPss and PS3 features formed more contributions to model than the other features. For H_990 and M_944, the RF model trained using PSTNPss features outperformed the other features. Whereas the LR model trained using PS3 features outperformed the other features for S_628. Overall, the contributions to the model performance of the six features were PSTNPss > PS3 > PS2 > binary > ENAC > NCP for *H. sapiens*, PS3 > PSTNPss > PS2 > binary > ENAC > NCP for *S. cerevisiae*, and PSTNPss > PS3 > ENAC > PS2 > binary > NCP for *M. musculus*. However, no single type of feature consistently outperformed other features for any species, and no single algorithm consistently outperformed other algorithms for any species. We can see that a single model using a single feature is unsatisfactory; therefore, we may need to integrate learning strategies to improve model performance.

In the experiment, we found that the PS3 features made a considerable contribution to the model performance, and the feature vector dimensions of PS3 were particularly high, up to more than 1000 dimensions. In theory, the more features, the more likely it is to provide features with strong discrimination ability in limited training samples. However, too many features may cause redundancy and “dimension disaster” (Suresh et al., 2022), which will lead to a long training time of the model and the risk of over-fitting, and reduce the generalisation ability of the model. Feature selection can remove some redundant features, reduce training time, select truly relevant features, and enhance the prediction performance of the model (Jones et al., 2021; Zhang et al., 2021; Suresh et al., 2022).

**FIGURE 3:**
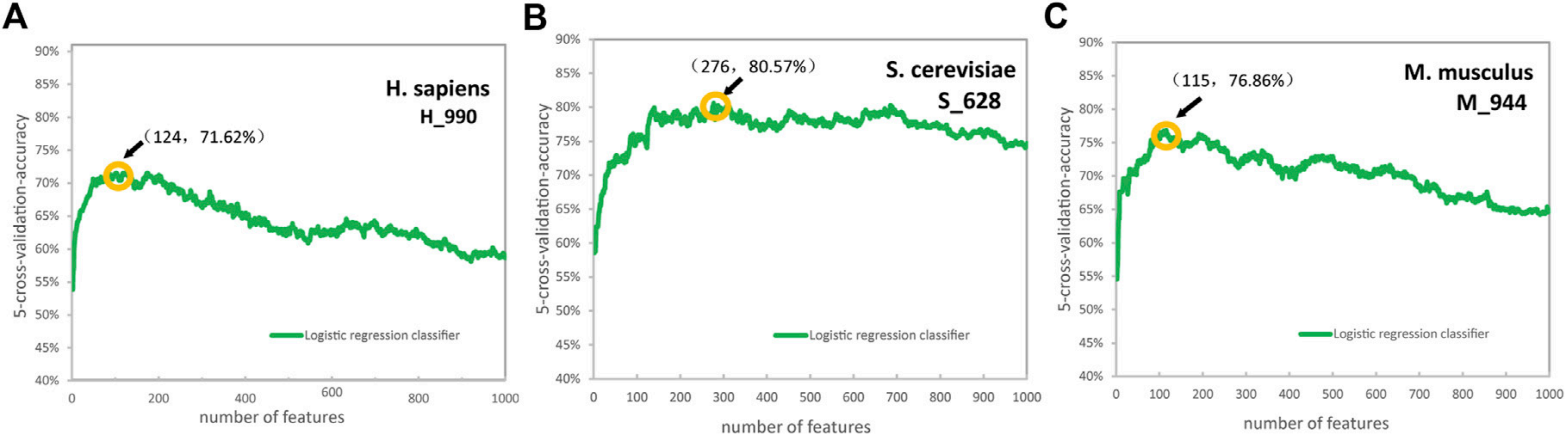
The accuracy curves for *H. sapiens* **(A)**, *S. cerevisiae* **(B)**, and *M. musculus* **(C)** of the position-specific of three nucleotides encoding schemes. (Due to the excessive dimensions of position-specific of three nucleotides features, 1000 features were selected for drawing for convenience).

Based on the LR algorithm, we employed a chi-square test and the IFS strategy to determine the optimal features (Dao et al., 2019; Lv et al., 2020; Zhang et al., 2021) were employed. We first ranked the feature importance of the six encoding schemes using a chi-square test separately, then set a whole ranked features set, named F: F = {f _1_, f _2_, ...f _n−1_, f _n_}, where *n* represent the features number. We tested the training dataset using the IFS by performing 5-fold cross-validation tests. In each iteration, IFS added a feature in F to the preliminary feature subset to build *n* feature subsets. When the highest ACC value was achieved, optimal feature subsets were obtained. The ACC curves for *H. sapiens*, *S. cerevisiae*, and *M. musculus* of PS3 encoding schemes are shown in Figure 3. When the number of features was the top 124, 276, and 115, we obtained the best predictive accuracies of 71.62%, 80.57%, and 76.86% for identifying Ψ sites in *H. sapiens*, *S. cerevisiae*, and *M. musculus*, respectively (Figure 3).

The ACC curves of the ENAC, binary, NCP, and PS2 encoding schemes are shown in Supplementary Figures S1–4. The optimal features are: the top 46 from 80 of ENAC, top 23 from 84 of binary, top 34 from 63 of NCP, and top 100 from 320 of PS2 for *H. sapiens*, the top 21 from 120 of ENAC, top 40 from 124 of binary, top 37 from 93 of NCP, and top 116 from 480 of PS2 for *S. cerevisiae*, and the top 17 from 80 of ENAC, top 49 from 84 of binary, top 44 from 63 of NCP, and top 63 from 320 of PS2 for *M. musculus*. The feature dimension of the PSTNPss is small; therefore, all PSTNPss features are selected.

Next, we examined the best combination of features. We used the first-ranked feature to build the PseU-ST model, added the second feature to build a new model, and then the third feature, until all of the obtained features were added. The performances of the feature combinations for *H. sapiens*, *S. cerevisiae*, and *M. musculus* are displayed in Supplementary Table S1. The optimal feature combination was PS3 + PSTNPss for *S. cerevisiae*, and that for *M. musculus* was PSTNPss + PS3, which both achieved the best performance of all metrics in either 5-fold cross-validation or independent testing (Supplementary Table S1). For *H. sapiens*, PSTNPss + PS3 achieved the best performance in 5-fold cross-validation, but the MCC and Sn of PSTNPss + PS3 + PS2 were better in independent testing, the ACC and Sp of PSTNPss + PS3 + PS2 + binary + ENAC were better in independent testing, but just 0.28%, 1.00%, 0.5%, and 7% higher, respectively. Therefore, PSTNPss + PS3 was selected as the optimal feature combination for *H. sapiens*.

### 3.2 Evaluation of the base-classifier combinations

**FIGURE 4:**
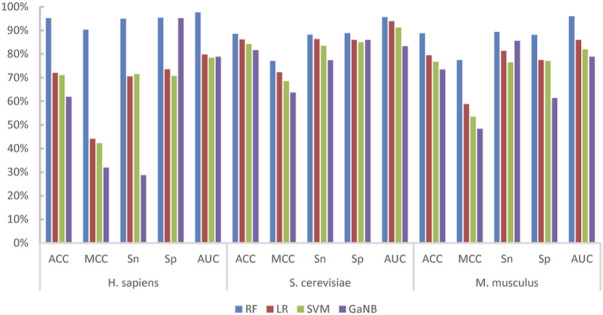
The performances of the four base-classifiers for *H. sapiens*, *S. cerevisiae*, and *M. musculus*.

We built integrated learning models using the stacking strategy. First, we built the RF, LR, SVM, and GaNB models separately as the candidate base classifier using the optimal feature combination selected in the forward step, namely, PSTNPss + PS3 for *H. sapiens*, PS3 + PSTNPss for *S. cerevisiae*, and PSTNPss + PS3 for *M. musculus*. We compared the performance of the four models for each species and ranked them according to ACC. The performances of the four models for each species are exhibited in Figure 4. The order of best performance the four models for each species was RF, LR, SVM, and GaNB (Figure 4). The performances of the RF models were good, but there was obvious over-fitting in *H. sapiens* and *S. cerevisiae*, so we employed the stacking strategy. We trained the stacked model using LR as the meta-classifier to determine the optimal base-classifiers. We assessed three different base-classifier combinations, which were RF + LR, RF + LR + SVM, and RF + LR + SVM + GaNB. The performances of the three combinations for each species is listed in Table 3. For *H. sapiens*, the combination of RF + LR achieved the best performance of all metrics in either cross validation or independent testing (Table 3). For *M. musculus*, the combination of RF + LR achieved the optimal performance of all metrics in cross validation too. For *S. cerevisiae*, the combination of RF + LR + SVM + GaNB achieved the best performance for almost all of the metrics in cross validation, but the performance of RF + LR had the best performance for all metrics in independent testing. Comparing the performance of the two combinations, it was found that in cross validation, the ACC, MCC, and Sp of RF + LR + SVM + GaNB were 0.32%, 0.64%, and 0.63% higher than those of RF + LR, but the AUC was lower by 0.78%, and the Sn was equal. In independent testing, the performance of RF + LR was better than that of RF + LR + SVM + GaNB in terms of all performance metrics, with ACC, MCC, Sn, Sp, and AUC being 2.00%, 4.00%, 2.00%, 2.00%, and 2.52% higher, respectively. Therefore, RF + LR was selected as the optimal base-classifier combination for *S. cerevisiae*.

**TABLE 3:** The performances of the base-classifier combinations for the three species.

Species	Base classifiers combination	5-Fold cross- validation					Independent testing				
		ACC (%)	MCC (%)	Sn (%)	Sp (%)<	AUC (%)	ACC (%)	MCC (%)	Sn (%)	Sp (%)	AUC (%)
*H. sapiens*	RF + LR	**93.64**	**87.28**	**94.34**	**92.93**	**98.56**	**89.00**	**79.02**	**97.00**	**81.00**	**96.51**
	RF + LR + SVM	93.43	86.88	**94.34**	92.53	98.42	86.50	73.84	94.00	79.00	95.47
	RF + LR + SVM + GaNB	92.93	85.88	93.94	91.92	98.41	86.00	74.17	**97.00**	74.00	95.56
*S. cerevisiae*	RF + LR	87.74	75.49	**86.94**	88.54	**95.95**	**83.50**	**67.00**	**83.00**	**84.00**	**89.00**
	RF + LR + SVM	87.74	75.49	**86.94**	88.54	95.25	82.50	65.00	82.00	83.00	87.64
	RF + LR + SVM + GaNB	**88.06**	**76.13**	**86.94**	**89.17**	95.17	81.50	63.00	81.00	82.00	86.48
*M. musculus*	RF + LR	**89.60**	**79.21**	**90.66**	**88.54**	**96.20**					
	RF + LR + SVM	87.47	74.96	88.32	86.62	95.29					
	RF + LR + SVM + GaNB	87.37	74.74	88.11	86.62	95.28					

Notes: Bold values indicate the best performance in terms of the corresponding measure.

**FIGURE 5:**
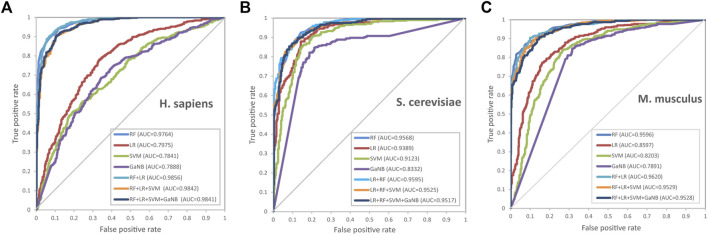
Receiver operating characteristic curves for the base classifiers and the stacked models of different base-classifier combinations during 5-fold cross-validation. The vertical coordinate is the true positive rate (sensitivity), while the horizontal coordinate is the false positive rate (1-specificity). [**(A)** *H. sapiens*, **(B)** *S. cerevisiae* and **(C)** *M. musculus*].

We further drew ROC curves to assess the performance of base classifiers and stacked models of different combinations. As seen in Figure 5, in cross validation, the combination of RF + LR reached the optimal performance of the AUC in all three species, *H. sapiens*, *S. cerevisiae*, and *M. musculus*, which is 98.56%, 95.95%, and 96.20%, respectively. Taken together, we selected RF + LR as the optimal base-classifier combination for the stacked model and named this stacked model PseU-ST.

### 3.3 Comparison with the other existing methods

To further examine the performance of PseU-ST, we compared it with other existing methods using the same benchmark training, listed in Tables 4, 5. As shown in Table 4, compared with other existing methods using the same training datasets, PseU-ST performed best in three important measures across all three species, that is, ACC, MCC, and Sn. For H_990, the ACC and MCC of PseU-ST were 13.94% and 27.28% higher, respectively, than those of the second-best method, iPseU-Layer. The Sn of PseU-ST was 5.23% higher than that of the second-best method, Porpoise. For S_628, the ACC, MCC, and Sn of PseU-ST were 6.05%, 12.11%, and 5.73% higher, respectively, than those of the second-best method, Porpoise. For M_944, the ACC, MCC, and Sn of PseU-ST were 0.26%, 0.21%, and 5.98% higher, respectively, than those of the second-best method, iPseU-Layer. In addition, for H_990, the Sp of PseU-ST was 4.71% higher than that of the second-best method, iPseU-Layer.

**TABLE 4:** Performance comparison of PseU-ST and other existing methods on the same benchmark training datasets.

Species	*H. sapiens*				*S. cerevisiae*				*M. musculus*			
Method	ACC (%)	MCC (%)	Sn (%)	Sp (%)	ACC (%)	MCC (%)	Sn (%)	Sp (%)	ACC (%)	MCC (%)	Sn (%)	Sp (%)
**PseU-ST**	**93.64**	**87.28**	**94.34**	**92.93**	**87.74**	**75.49**	**86.94**	**88.54**	**89.60**	**79.21**	**90.66**	**88.54**
PseUdeep	66.99	35.00	74.47	60.71	72.73	45.00	61.75	78.13	72.45	44.00	66.70	77.36
PsoEL-PseU	70.80	42.00	66.90	74.70	80.30	62.00	69.10	91.40	76.50	53.00	82.20	70.80
Porpoise	78.53	58.45	89.11	67.94	81.69	63.38	81.21	82.17	77.75	55.55	77.83	77.67
iPseU-Layer	79.70	60.00	71.18	88.22	80.08	60.00	77.92	81.82	89.34	79.00	84.68	93.76
RF-PseU (10-fold)	64.30	29.00	66.10	62.60	74.80	49.00	77.20	72.40	74.80	50.00	73.10	76.50
RF-PseU (LOO)	64.00	29.00	65.90	62.60	75.80	52.00	78.20	73.40	74.50	48.00	72.70	75.20
EnsemPseU	66.28	33.00	63.46	69.09	74.16	49.00	73.88	74.45	73.85	48.00	75.43	72.25
XG-PseU	65.44	31.00	63.64	67.24	68.15	37.00	66.84	69.45	72.03	45.00	76.48	67.57
iPseU-CNN	66.68	34.00	65.00	68.78	68.15	37.00	66.36	70.45	71.81	44.00	74.79	69.11
PseUI	64.24	28.00	64.85	63.64	65.13	30.00	62.74	67.52	70.44	41.00	74.58	66.31
iRNA-PseU	60.40	21.00	61.01	59.80	64.49	29.00	64.65	64.33	69.07	38.00	73.31	64.83

Notes: 10-fold–10-fold cross-validation; LOO—leave-one-out cross-validation. Bold values indicate the performance of PseU-ST.

**TABLE 5:** Performance comparison of PseU-ST and other existing methods on the same independent test datasets.

Species	*H. sapiens*				*S. cerevisiae*			
Method	ACC (%)	MCC (%)	Sn (%)	Sp (%)	ACC (%)	MCC (%)	Sn (%)	Sp (%)
**PseU-ST**	**89.00**	**79.02**	**97.00**	**81.00**	**83.50**	**67.00**	**83.00**	**84.00**
PseUdeep	66.18	33.00	73.53	58.82	80.88	62.00	77.45	84.31
PsoEL-PseU	75.50	51.00	76.00	75.00	82.00	64.00	83.00	81.00
Porpoise	77.35	55.13	82.30	72.40	83.50	67.27	88.00	79.00
iPseU-Layer	71.00	43.00	63.00	79.00	72.50	45.00	68.00	77.00
RF-PseU (10-fold)	75.00	50.00	78.00	72.00	77.00	54.00	75.00	79.00
RF-PseU (LOO)	74.00	48.00	74.00	74.00	74.50	49.00	70.00	79.00
EnsemPseU	69.50	39.00	73.00	66.00	75.00	51.00	85.00	65.00
XG-PseU	67.50	35.00	68.00	67.00	71.00	42.14	75.00	67.00
iPseU-CNN	69.00	40.00	77.72	60.81	73.50	47.00	68.76	77.82
PseUI	65.50	31.00	64.85	68.00	68.50	37.00	65.00	72.00
iRNA-PseU	61.50	23.00	58.00	65.00	60.00	20.00	63.00	57.00

Notes: 10-fold–10-fold cross-validation; LOO—leave-one-out cross-validation. Bold values indicate the performance of PseU-ST.

To examine if PseU-ST models are subjected to over-fitting, we performed independent testing on independent test datasets to validate the models. The performance comparison of PseU-ST and other existing methods is presented in Table 5. As indicated, PseU-ST performed the best in all four measures for H_200. The ACC, MCC, and Sn of PseU-ST was 11.65%, 23.89%, and 14.70% higher, respectively, than those of the second-best method, Porpoise, and the Sp of PseU-ST was 2.00% higher than that of the second-best method, iPseU-Layer.

Besides, there was little difference between the prediction performance of independent and cross validation tests, for instance, the ACC and MCC of PseU-ST on H_200 was 89.00% and 79.02%, respectively, which is close to those of H_990 (93.64% and 87.28%, respectively). PseU-ST obtained an ACC of 83.5% and MCC of 67.00% on S_200, which are also very close to those of S_628 (87.74% and 75.49%, respectively), and there was no over-fitting.

In summary, compared with other existing models, PseU-ST achieved better prediction performance and had obvious advantages. PseU-ST is a highly competitive model for identifying RNA Ψ sites in *H. sapiens*, *S. cerevisiae*, and *M. musculus*.

### 3.4 The interpretation of model

**FIGURE 6:**
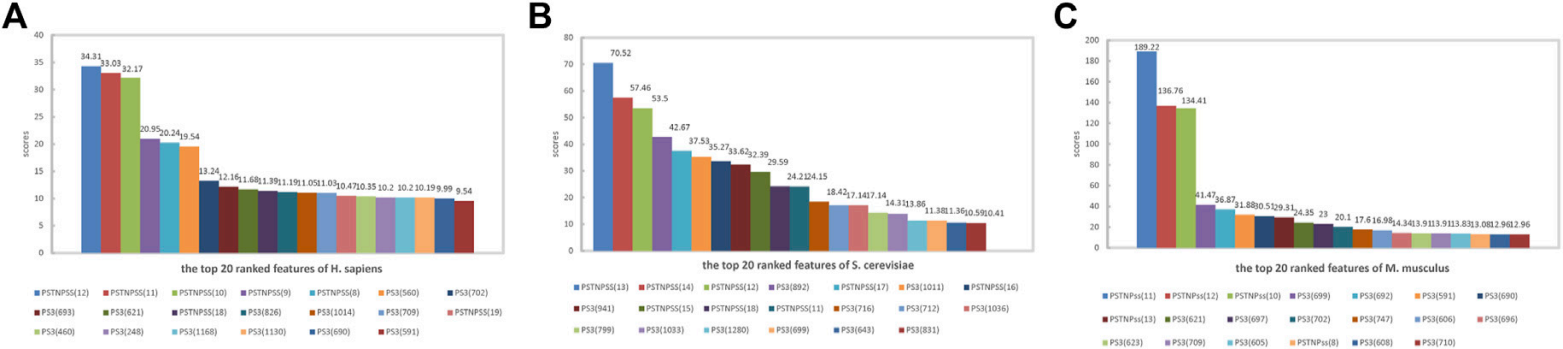
Top 20 features of PseU-ST ranked according to feature scores for predicting RNA Ψ sites of **(A)** *H. sapiens*, **(B)** *S. cerevisiae*, and **(C)** *M. musculus*.

To interpret the feature importance for the performance of the PseU-ST models. We ranked the features in the model of all three species according to feature scores and mapped the top 20 ranked features of each species in Figure 6. The PSTNPss features played an important role in the PseU-ST models; the top three important features for all three species models were PSTNPss features, and their scores were significantly higher than those of other features (Figure 6). This indicates that the PSTNP features plays a crucial role in PseU-ST models and makes more contributions to the performance of PseU-ST. Owing to the large proportion of PS3 features in the PseU-ST models, the contribution of these features to the prediction performance cannot be ignored.

## 4 Conclusion

In our study, a novel stacked ensemble-learning method named PseU-ST (available at https://github.com/jluzhangxinrubio/PseU-ST) was developed to identify RNA Ψ sites in *H. sapiens, S. cerevisiae*, and *M. musculus* with a more stable and accurate performance. We thoroughly evaluated almost all of the RNA sequence encoding schemes available in the iLearnPlus software package and tested seven most popular machine learning algorithms to determine the optimal feature and best base-classifier combinations. Finally, we developed an optimised model for each of the three species. Owing to the adoption of a stacking strategy and the employ of optimal feature selection algorithms, PseU-ST achieved better performance on either cross-validation or independent tests compared with the other existing models. In addition, we interpreted the feature importance for the PseU-ST models, in which PSTNPss features were shown to play an important role.

The strategies used in this study are universal and they can be employed to predict other DNA/RNA modification sites, such as DNA N4-methylcytosine and 5-methylcytosine sites. We believe PseU-ST will be a powerful tool for promoting a community-wide works for identifying Ψ sites and supplying high-quality identified Ψ sites for biological validation.

## Data Availability

The original contributions presented in the study are included in the article/Supplementary Material, further inquiries can be directed to the corresponding author.

## Abbreviations

Accuracy, (ACC); Adaptive Boosting, (AdaBoost); Area under the receiver operating curve, (AUC); Convolutional neural network, (CNN); Enhanced nucleic acid composition, (ENAC); eXtreme Gradient Boosting, (XGBoost); Gaussian Naive Bayes, (GaNB); Incremental feature selection, (IFS); k-nearest neighbour, (KNN); Light gradient boosting machine, (lightGBM); Logistic regression, (LR); Matthew’s Correlation Coefficient, (MCC); Naïve Bayes, (NB); Nucleotide chemical property, (NCP); Position-specific of three nucleotides, (PS3); Position-specific of two nucleotides, (PS2); Position-specific trinucleotide propensity based on single-strand, (PSTNPss); Random forest, (RF); Receiver Operating Characteristic, (ROC); Sensitivity, (Sn); Specificity, (Sp); Support vector machine, (SVM).
