# Supplementary material for: PseU-ST: A new stacked ensemble-learning method for identifying RNA pseudouridine sites
Source: Front Genet. 2023 Jan 19;14:1121694. doi: 10.3389/fgene.2023.1121694 (PMC9892456; doi:10.3389/fgene.2023.1121694)
Supplement: Supplementary file 1 [file DataSheet1.pdf]

Supplementary Material

PseU-ST: a new stacked ensemble-learning method for identifying RNA pseudouridine sites

Xinru Zhang, Shutao Wang, Lina Xie, Yuhui Zhu*

*** Correspondence:** Yuhui Zhu zhuyuh@jlu.edu.cn

1 Supplementary Figures

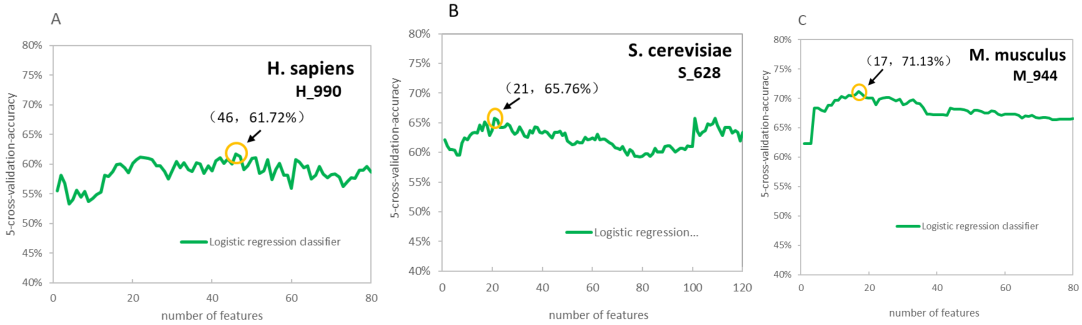

Figure S1 The ACC curves for H. sapiens(A), S. cerevisiae(B) and M. musculus(C) of the ENAC encoding schemes

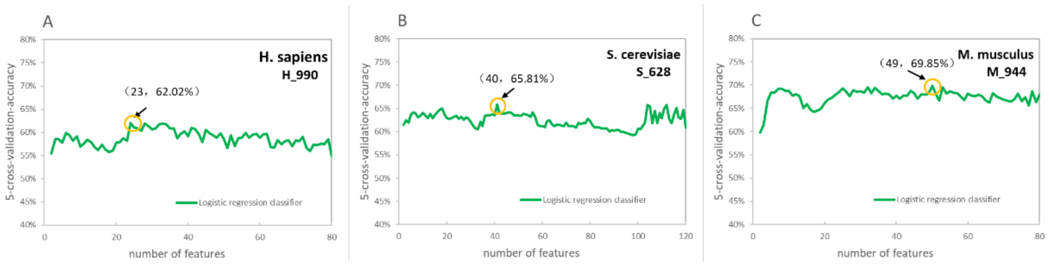

Figure S2 The ACC curves for H. sapiens(A), S. cerevisiae(B) and M. musculus(C) of the Binary encoding schemes

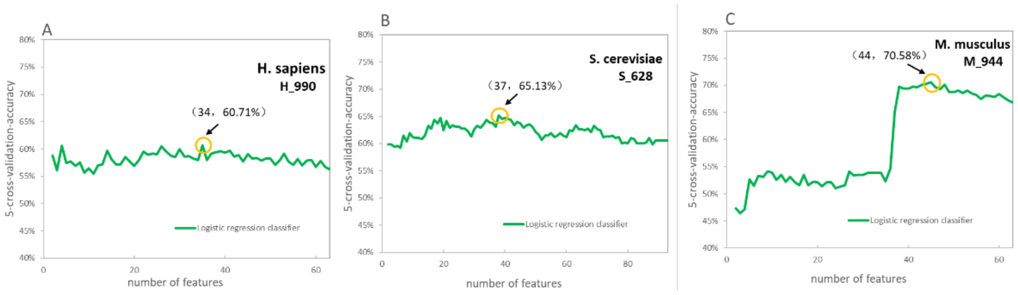

Figure S3 The ACC curves for H. sapiens(A), S. cerevisiae(B) and M. musculus(C) of the NCP encoding schemes

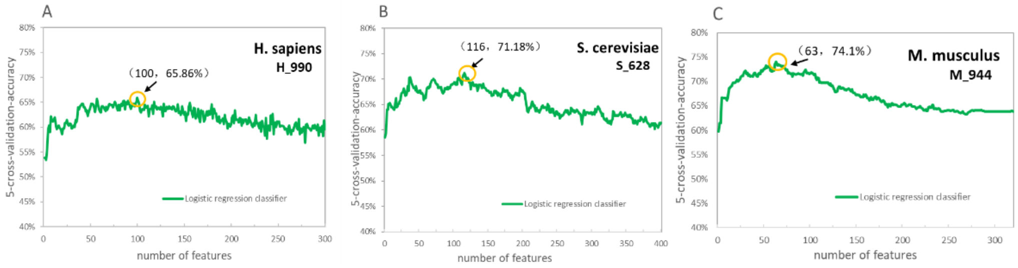

Figure S4 The ACC curves for H. sapiens(A), S. cerevisiae(B) and M. musculus(C) of the PS2 encoding schemes

Table S1. The performances of the feature combinations for the three species

Species	Feature combination	5-fold cross- validation					Independent testing				
		ACC (%)	MCC (%)	Sn (%)	Sp (%)	AUC (%)	ACC (%)	MCC (%)	Sn (%)	Sp (%)	AUC (%)
H. sapiens	PSTNPss	89.29	78.70	87.77	90.95	93.70	73.00	46.46	80.00	66.00	70.18
	PSTNPss + PS3	**93.64**	**87.28**	**94.34**	**92.93**	**98.56**	89.00	79.02	97.00	81.00	**96.51**
	PSTNPss + PS3 + PS2	87.07	74.17	88.48	85.66	95.52	89.00	**79.30**	**98.00**	80.00	94.99
	PSTNPss + PS3 + PS2 + binary	84.44	68.91	85.66	83.23	94.35	87.00	74.24	91.00	83.00	94.36

	PSTNPss + PS3 + PS2 + binary + ENAC	83.43	66.89	84.65	82.22	93.42	**89.50**	79.04	91.00	**88.00**	95.51
	PSTNPss + PS3 + PS2 + binary + ENAC + NCP	82.02	64.07	83.43	80.61	92.33	89.00	78.39	94.00	84.00	95.20
S. cerevisiae	PS3	78.34	56.69	78.66	78.03	86.48	62.00	24.00	61.00	63.00	73.89
	PS3 + PSTNPss	**87.74**	**75.49**	**86.94**	**88.54**	**95.95**	**83.50**	**67.00**	**83.00**	**84.00**	**89.00**
	PS3 + PSTNPss + PS2	85.67	71.36	84.39	86.94	95.33	82.50	65.03	81.00	84.00	88.97
	PS3 + PSTNPss + PS2 + binary	85.99	72.05	83.76	88.22	94.34	82.00	64.05	80.00	84.00	89.49
	PS3 + PSTNPss + PS2 + binary + ENAC	85.19	70.49	82.48	87.90	94.01	82.50	65.03	81.00	84.00	89.74
	PS3 + PSTNPss + PS2 + binary + ENAC + NCP	85.03	70.11	83.12	86.94	93.50	81.50	63.08	79.00	84.00	90.06
M. musculus	PSTNPss	82.34	64.60	82.81	84.30	90.21					

	PSTNPss + PS3	**89.60**	**79.21**	**90.66**	**88.54**	**96.20**					
	PSTNPss + PS3 + ENAC	88.22	76.43	88.11	88.32	95.27					
	PSTNPss + PS3 + ENAC + PS2	87.90	75.81	88.75	87.05	94.88					
	PSTNPss + PS3 + ENAC + PS2 + binary	86.31	72.62	87.05	85.56	94.19					
	PSTNPss + PS3 + ENAC + PS2 + binary + NCP	85.46	70.92	86.20	84.71	93.71					

Notes: Bold values indicate the best performance in terms of the corresponding measure.
